# Zeaxanthin-Rich Extract from Superfood *Lycium barbarum* Selectively Modulates the Cellular Adhesion and MAPK Signaling in Melanoma versus Normal Skin Cells In Vitro

**DOI:** 10.3390/molecules26020333

**Published:** 2021-01-11

**Authors:** Diana Cenariu, Eva Fischer-Fodor, Adrian Bogdan Țigu, Andrea Bunea, Piroska Virág, Maria Perde-Schrepler, Vlad-Alexandru Toma, Andrei Mocan, Ioana Berindan-Neagoe, Adela Pintea, Gianina Crișan, Mihai Cenariu, Alma Maniu

**Affiliations:** 1 Medfuture Research Center for Advanced Medicine, University of Medicine and Pharmacy, “Iuliu Hatieganu”, RO-400012 Cluj-Napoca, Romania; diana.cenariu@umfcluj.ro (D.C.); fischer.eva@iocn.ro (E.F.-F.); adrianbogdantigu@gmail.com (A.B.Ț.); 2 Institute of Oncology “I. Chiricuta”, 34-36 Republicii Street, RO-400015 Cluj-Napoca, Romania; vpiroska@yahoo.com (P.V.); pmariaida@yahoo.com (M.P.-S.); ioananeagoe29@gmail.com (I.B.-N.); 3 Faculty of Biology and Geology, Babeș-Bolyai University, RO-400015 Cluj-Napoca, Romania; vlad.al.toma@gmail.com; 4 Department of Chemistry and Biochemistry, University of Agricultural Sciences and Veterinary Medicine, RO-400372 Cluj-Napoca, Romania; andrea.bunea@usamvcluj.ro (A.B.); apintea@usamvcluj.ro (A.P.); 5 Institute of Biological Research Cluj-Napoca, Branch of NIRDBS Bucharest, 400015 Cluj-Napoca, Romania; 6 National Institute for Research and Development of Isotopic and Molecular Technologies, RO-400293 Cluj-Napoca, Romania; 7 Department of Pharmaceutical Botany, University of Medicine and Pharmacy “Iuliu Hatieganu”, RO-400337 Cluj-Napoca, Romania; gcrisan@umfcluj.ro; 8 Laboratory of Chromatography-ICHAT, University of Agricultural Sciences and Veterinary Medicine, RO-400372 Cluj-Napoca, Romania; 9 Research Center for Functional Genomics, Biomedicine and Translational Medicine, “Iuliu Hatieganu”, University of Medicine and Pharmacy, RO-400012 Cluj-Napoca, Romania; 10 Biotechnology Research Center, University of Agricultural Sciences and Veterinary Medicine, RO-400372 Cluj-Napoca, Romania; 11 Department of Otolaryngology, University of Medicine and Pharmacy “Iuliu Hatieganu”, RO-400372 Cluj-Napoca, Romania; aurelia.maniu@umfcluj.ro

**Keywords:** zeaxanthin, Goji, normal fibroblast, malignant melanoma, CD105, CD44, MAPK, ERK, JNK, p38

## Abstract

The concern for implementing bioactive nutraceuticals in antioxidant-related therapies is of great importance for skin homeostasis in benign or malignant diseases. In order to elucidate some novel insights of *Lycium barbarum* (Goji berry) activity on skin cells, the present study focused on its active compound zeaxanthin. By targeting the stemness markers CD44 and CD105, with deep implications in skin oxidative stress mechanisms, we revealed, for the first time, selectivity in zeaxanthin activity. When applied in vitro on BJ human fibroblast cell line versus the A375 malignant melanoma cells, despite the moderate cytotoxicity, the zeaxanthin-rich extracts **1** and **2** were able to downregulate significantly the CD44 and CD105 membrane expression and extracellular secretion in A375, and to upregulate them in BJ cells. At mechanistic level, the present study is the first to demonstrate that the zeaxanthin-rich Goji extracts are able to influence selectively the mitogen-activated protein kinases (MAPK): ERK, JNK and p38 in normal BJ versus tumor-derived A375 skin cells. These results point out towards the applications of zeaxanthin from *L. barbarum* as a cytoprotective agent in normal skin and raises questions about its use as an antitumor prodrug alone or in combination with standard therapy.

## 1. Introduction

Food intake of bioactive antioxidant nutraceuticals enables skin cells protection and it influences malignant epithelial tumor growth. The concern for implementing nutraceuticals in antioxidant-related therapies is of great importance in prevention of skin homeostasis instability and occurrence of malignant illness. Many antioxidants extracted from natural sources are suitable for topical applications against various skin disorders, and beyond the topical applications, the natural extracts intake as from food can maintain the skin health condition. Over 700 carotenoids can be found in nature but only few of them have nutritional value and are present in the human body: the provitamin A carotenoids (e.g., β-carotene) and the non-provitamin A carotenoids (lycopene, zeaxanthin and its stereoisomer lutein). The antioxidant carotenoids zeaxanthin and lutein are xanthophylls, lipophilic pigments derived mainly from plant sources in the diet which the human body cannot synthesize [1]. They have free hydroxyl groups at each end of the molecule that provide unique biochemical properties that allow them to orient within cell membranes [2], quenching singlet molecular oxygen and other reactive oxygen species (ROS) [3]. Zeaxanthin supplementations provide health benefits to normal skin by modulating the expression of several genes [4] and fight against the formation of ROS [5], acting as filters for UV light exposure by accumulation in high concentrations in skin tissue. Instead, the melanoma cells growth and migration are hindered by zeaxanthin [6]. Previous experiments [7] demonstrated that the activity of zeaxanthin at 1 μM was not significantly different from the controls, but at higher concentrations it manifested a cytotoxic effect on cultured human uveal melanoma cells (3–10 μM) and respectively on BJ cells proliferation (100–300 μM). Zeaxanthin at high dosage of 10.0 μM lowered NF-kβ levels due to the inhibition of NF- kβ pathway in uveal melanoma cells [7].

Goji berries (*Lycium barbarum*, *Solanaceae*), a traditional Asian food and medicine, are a rich source of zeaxanthin. *L. barbarum* became very popular during the past two decades due to its proven nourishing value, anti-inflammatory and antiaging effects, and its important role in prevention and cure of various chronical diseases. Goji berries contain many nutrients with high biological activity, such as carotenoids (0.03%–0.5% of the dry matter, with zeaxanthin dipalmitate representing up to 80% of the total carotenoid), polysaccharide complexes (5%–8% of the total dry matter of the fruits), phenylpropanoids, flavonoid fractions, vitamins and fatty acids [8]. Goji antioxidative activity is mainly attributed to its carotenoid content [8,9]. Many research studies demonstrated *L. barbarum* extracts immunomodulation [10,11], antitumor activity [12,13,14] and skin protection from UV radiation [15]. The clinical efficacy of Goji berry is not yet established but its pharmaceutical properties in vitro and in vivo suggest that it may be beneficial in the prevention and treatment of tumors [16]. Carotenoids from *L. barbarum* fruits are associated with the extracts antitumor effect [17]. The anti-inflammatory effect was tested on mice that consumed *L. barbarum* juice, and the results showed a significant effect on basal and stimulated cytokine production [18,19] In human skin, *L. barbarum* has the ability to influence the matrix metalloproteinase, which can lead to the tissues protection [20].

The mitogen activated protein kinase (MAPK) family is composed of three types of protein kinases: extracellular responsive kinase (ERK), the stress-activated c-Jun N-terminal kinases (JNK) and p38 kinase family. The three types of MAPK implicated in the control of apoptosis and their interaction, mainly a balance between ERK and the activity of stress kinases, may dictate if a cell will survive or undertake death pathways [21]. In dermal fibroblasts, zeaxanthin is able to modulate the intracellular expression of MAP kinases such Erk 1/2 of p38 [6]. The in vivo experiment performed by Xiao J. et al. [22] on the influence of zeaxanthin dipalmitate on fatty liver diseases of rats demonstrated expressional changes of key MAPK members: p38, MAPK and ERK1/2 including modulation of NF-kβ activity, but had no influence on JNK. They concluded that treatment with zeaxanthin expressed hepatoprotective, anti-inflammatory, anti-oxidative and anti-apoptotic properties. Conversely, the non-kinase mediated pathways are likewise important in melanoma cells progression, such the non-kinase membrane glycoprotein CD44 activation [23]. The mesenchymal membrane marker CD44 and the adhesion-related protein CD105 (or endoglin) are important markers of stemness in skin-derived fibroblasts and in melanoma cells [24,25,26]; the malignant melanoma is abundant in CD44 and CD105, including the melanoma-initiating cells [27], which confer aggressivity to the tumor growth. CD44 regulates the migration of fibroblast and can enhance as well abnormal epidermal function and melanoma development [28]. The crosstalk of the two adhesion molecules CD44 and CD105 with the MAPK pathway has been proven [29].

When Goji berries’ biologic effect was studied in normal skin or melanoma, generally in the spotlight were nutrients such polysaccharides, and only rarely zeaxanthin or its palmitate [30]. Meanwhile, for *Lycium barbarum*-derived polysaccharides, several molecular targets were already identified [31]; the mechanisms of action attributed to zeaxanthin isolated from the same extracts is unknown.

The bioavailability of zeaxanthin from food intake is dependent on its metabolism, including the esterification, and this process influences the accumulation of zeaxanthin in eyes, liver, intestines, blood vessels and in skin [1]. Several data strongly suggest that zeaxanthin supplementations may have a protective role in normal skin [1]; further, there are a few studies that address *L. barbarum*/Goji berries’ biologic outcome on malignant melanoma of skin. Huang and co-workers (2014), in a comprehensive paper, studied another *Lycium* variety (*L. chinense*) and the chosen biologic system was a murine melanoma cell line, not a human one. There are many pending questions [1] that have led us to study the mechanisms which underlie to the effect of zeaxanthin and its metabolites in the human skin.

Therefore, in the present paper, we proposed to study the effect of zeaxanthin-rich extract from two distinct *Lycium barbarum* (Goji berry) varieties: Erma (**1**) and Biglifeberry (**2**), and to elucidate if they act selectively against normal skin cells versus malignant melanoma-derived cells. For the remainder of the manuscript, the term extract **1** or **2** will be employed when referring to zeaxanthin-rich Goji berry extracts Erma (**1**) and Biglifeberry (**2**).

Cytotoxicity of extracts **1** and **2** was measured, as well as the capacity of **1** and **2** to modulate the cells reducing potential and the NF-kβ transcription factor. The effect of **1** and **2** on CD44 and CD105 expression was assessed at unicellular level; these results were validated through the assessment of the treated cells extracellular secretory function. At the mechanistic level, the present manuscript presents the novelty by highlighting the Goji-derived zeaxanthin effects on the ROS-signal mediator protein kinases ERK, JNK and p38 in parallel in melanoma cells and normal fibroblasts. To elucidate new targets of carotenoid-rich Goji extracts, we focused on CD44 and CD105 membrane markers, two molecules expressed on human dermal fibroblasts and as well on melanoma cells, with deep implications in redox signaling and stemness.

## 2. Materials and Methods

### 2.1. Plant Material

Goji “Erma” and “Biglifeberry” and are two varieties of *Lycium barbarum*, known for their high antioxidant content. Fruits of *L. barbarum* L. from two cultivars were collected in the summer of 2014 from two origins: cultivar Erma (**1**) was collected from an ecological culture in North-Vest Romania, Ciuperceni (47°52′14″ N, 23°0′55″ E), Satu-Mare County; cultivar Biglifeberry (**2**) was collected from an ecological culture in NV Romania, Ploscoș (46°38′33″ N, 23°50′43″ E), Cluj County [32]. The fruit were harvested at full maturity and stored at −20 °C until analysis.

### 2.2. Isolation of Carotenoids from L. barbarum Varieties Erma and Biglifeberry

Frozen Goji berries (20 g) were homogenized with 2 g of sodium bicarbonate and repeatedly extracted with methanol/ethyl acetate/petroleum ether (1:1:1, *v*/*v*/*v* to color exhaustion) [33]. The combined extracts were partitioned in a separation funnel with water, diethyl ether and saturated saline solution. The organic phase (diethyl ether and ethyl acetate) containing the pigments was filtered through anhydrous sodium sulphate and evaporated to dryness at 35 °C under vacuum, using a rotatory evaporator. The residue was dissolved in diethyl ether. A part of the total carotenoid extract was evaporated to dry (unsaponified extract) and another part was saponified with an equal volume of potassium hydroxide solution (30% in methanol), at room temperature for 5 h, under dimmed light [34]. The saponified extract was transferred into a separation funnel containing diethyl ether and washed with water until free of alkali. The solvent was completely evaporated and the saponified dry extract was further used for cell culture treatment. Fractions of both dry extracts (saponified and unsaponified) were dissolved in HPLC grade ethyl acetate and filtered through a 0.2 μm PTFE filter into amber glass for HPLC analysis. All experiments were performed under subdued light.

### 2.3. Cell Lines

The human normal fibroblast cell line was BJ HEP (code CRL-2522), from American Type Culture Collection (ATCC) acquired through LGC Standards GmbH, Wesel, Germany. The cells were cultivated in Eagle’s Minimal Essential Medium (MEM), supplemented with 10% fetal calf serum (FCS). The malignant melanoma cell line, A375 was from European Collection of Authenticated Cell Cultures (ECACC), through Sigma Aldrich Company, St. Louis, MO, USA. A375 cell line was cultivated in Dulbecco’s Modified Eagle’s medium (DMEM), supplemented as well with 10% FCS. All media and supplements were from Sigma Aldrich Company. The general instrumentation for in vitro testing were: Class II laminary hoods Lamil Plus 13 from Karstulan Metalli Oy, Karstula, Finland; 32R centrifuge with spin-out rotor from Hettich Lab Technology, Tuttlingen, Germany; Observer D.1 inverted phase fluorescence microscope from Carl Zeiss (Jena, Germany); Heto Ultrafreezer (Heto Holten, Allerd, Denmark), Cryosystem 2000 liquid nitrogen locator (MVE Bio-Medical Division, Burnsville, MN, USA).

### 2.4. Cytotoxicity Testing

To measure the zeaxanthin-rich Goji extracts’ biologic effects, the dilution of the dried saponified extracts was made with tetrahydrofuran (THF p.a, from Merck, Darmstadt, Germany). The concentration of **1** and **2** was normalized to obtain a stock solution of 10 mM zeaxanthin. Serial dilutions were made in phosphate buffered saline solution (PBS, from Sigma Aldrich) to obtain 2.5 to 250 μM final concentration in the cell culture media, considering that in each well were added 2 × 10^4^ cells in 190 μL cell culture media and 10 μL compound. Three separate wells were treated with each concentration on each cell line. The experiments were repeated three times. The untreated, reference wells were treated with PBS only.

The cytotoxicity of **1** and **2** was measured in triplicate, as described before [35], using the MTT dye (3-(4,5-dimethylthiazol-2-yl)-2,5-diphenyltetrazolium bromide, from Sigma Aldrich), which is transformed into its insoluble formazan form by the mitochondrial oxidoreductase enzymes, only in viable cells. The formazan crystals were solubilized in dimethyl sulfoxide (from Titolchimica, Pontecchio Polesine, Italy), and the 96-well plates were measured in colorimetry using a Synergy 2.0 microplate reader (from BioTek Company, Winooski, VT, USA) at 570 nm wavelength. The absorbance of each well reflects the number of viable cells present at the moment of the measurement.

### 2.5. Antiproliferative Capacity

The quantitative evaluation of **1** and **2** antiproliferative capacity was made using the Alamar Blue Cell Viability Reagent (from Molecular Probes, Eugene, OR, USA, acquired through Thermo Scientific Company), an indicator that uses the natural reducing power of living cells to convert resazurin to its fluorescent resorufin form. Following an earlier described method [36], we measured the dynamics of changes in reducing potential; three different time points were studied: 6-, 12- and 24-h, and each separate plate was processed. The BJ and A375 cells were plated on black Costar 96-well plates with clear bottom (from Corning BV, Amsterdam, Netherlands), at a 2 × 10^4^ cell/well density in 95 μL media. The adhered cells were treated with 5 μL of **1** and **2**, the same concentrations as for the MTT test. Three separate wells were treated with the same concentration for each compound. After the incubation, Alamar Blue reagent was added to each well and fluorescence was measured at 620 nm wavelength (excitation at 540 nm) using the Synergy 2.0 reader. Two independent experiments were made on both cell lines.

### 2.6. Flow-Cytometry Measurements of Membrane and Intracellular Markers

To identify the membrane markers, anti-human FITC conjugated anti-CD44 and anti-CD105 antibodies were used, both from Miltenyi Biotech (Bergisch Gladbach, Germany). The BJ and A375 cells were plated on 6-well plates (2.5 × 10^5^ cells/3 mL cell culture media), treated for 24 h with **1** and **2** at a subcytotoxic concentration of 50 μM, all samples in duplicates. As reference, untreated cells were growth in the same conditions; instead of Goji extracts, sterile PBS was added to these wells. The cells were harvested, washed with CellWash buffer (from BD Biosciences, San Jose, CA, USA), and 5 × 10^5^ cells were re-suspended in 80 μL PBS with 0.5% FCS. The appropriate amount of antibodies was added, according to the manufacturer’s indication, the cell suspensions were incubated, washed and prepared for flow-cytometry measurement, as described earlier [37].

To evaluate the intracellular protein expression, we used the goat anti-human p65 NF-kβ antibody (acquired from R&D Systems Europe Ltd., Abingdon, UK) with the appropriate PE anti-goat secondary antibody; goat anti-human phosphorilated ERK1+ERK2 (pT202/pY204 and pT185/pY187), phospho p38 (pT180/Y182) and phosphorilated JNK1/2 (pT183/Y185) (all from Abcam, Cambridge, UK). The cells were cultivated and treated as above, and after washing with cold PBS, the cells were permeabilized using the Inside Stain kit from Miltenyi Biotech. Two independent samples were prepared for each treated cell population. The samples were analyzed by flow cytometry, with a FACS Canto II Flow cytometer (from BD Biosciences, San Jose, CA, USA) using the 488-nm, blue, aircooled, 20-mW solid state excitation laser and the 530/30 filter for FITC as well as the 585/42 filter for PE.

### 2.7. Immunoenzymatic Testing (ELISA)

The intracellular soluble NF-kβ, and the extracellular, soluble CD105 and CD44 were quantitatively evaluated with the ELISA immunoenzymatic method. The BJ and A375 cells were processed as above (2.7), and separate wells were treated with 50 μM solution of **1** and **2,** in duplicates, for 6-, 12- and 24-h exposure.

The cell culture supernates were harvested from each well, aliquoted and kept at −80 °C in an ultra freezer. After thawing, the samples were centrifuged at 10,000 rpm for 5 min and the supernates were used. We used the human CD105 (ab100507) and human CD44 ELISA kits (ab45912) from Abcam, Cambridge, UK, according to the manufacturers’ indication. CD44 measurement: 100 µL of each sample and standard solution were added to pre-treated 96-well plate with a monoclonal antibody specific for CD44. For each treatment, two separate wells were filled, using supernates derived from parallel experiments. After 1 h of incubation at room temperature, three washing steps were performed using the 300 µL of Wash Buffer. An amount of 50 µL of 1X Biotinylated anti-CD44 was added to each well and 30 min incubation at room temperature was performed. After another three washing steps with 300 µL of Wash Buffer, 100 µL of 1X Streptavidin-HRP solution was added to each well. 100 µL of Chromogen TMB substrate solution was added to each well after three washing steps and the 96-well plate was incubated for 20 min at room temperature. An amount of 100 µL of Stop Reagent were added to each well and the measurements were performed immediately at 450 nm with a reference wavelength of 620 nm.

For CD105 assessment, 100 µL of each sample and standard solution were added to pre-treated 96-well plate with antibody specific for Human CD105, followed by a 2.5 h incubation step at room temperature. Four washing steps were performed with 300 µL of Wash Buffer. A 1 h incubation step, with gentle shaking at room temperature, was performed with 100 µL of 1X Biotinylated CD105 Detection Antibody. Another four washing steps were performed. After washing, a 45 min incubation step, with gentle shaking at room temperature, was performed after adding 100 µL of 1X HRP-Streptavidin solution. Four washing steps were performed, followed by 30 min incubation step, with gentle shaking at room temperature, with 100 µL of TMB One-Step Substrate Reagent. An amount of 50 µL of Stop Solution was added to each well and the measurements were performed immediately at 450 nm.

The intracellular NF-kβ was measured for three different concentrations: 25 μM, 50 μM and 100 μM after 24 h exposure. The adhered cells were washed with PBS, harvested and subjected to lysis, with the cell lysis buffer provided by the assay kit (ab176647- NF-kβ p65 SimpleStep ELISA kit from Abcam). The lysates were centrifuged, the protein level from each sample was quantified with the Bradford technique, as described elsewhere [38], and the protein concentrations of ELISA samples were normalized by dilution, in order to obtain identical concentration in each probe. The protocol was carried out according to the manufacturer’s indications. An amount of 50 µL of each sample was added to the pre-treated 96-well plate. Over each sample, in duplicate, a standard solution 50 µL of antibody cocktail was added, which contains the capture antibody and the detection antibody. After 1 h incubation at room temperature and 200 rpm shake, three automatic washing steps were performed using 350 µL of 1X washing buffer. An amount of 100 µL of TMB Substrate was added to the 96-well plate and a 15 min incubation step was performed on 200 rpm at dark. An amount of 100 µL of Stop Solution was added to each well that contains sample and standard solution and the measurement was performed at 450 nm on TECAN Sunrise ELISA plate reader with Magellan software (Tecan Group, Männedorf, Switzerland). The samples were in duplicates, and as reference we used untreated cells; the blank value was the cell culture media. For quantitative assessment, a standard curve was obtained from the measurements on standard NF-kβ p65 protein provided by manufacturer, and the single concentration values were calculated.

### 2.8. Data Analysis

The experimental results were analyzed using the GraphPad Prism5 biostatistics program (from GraphPad Software, La Jolla, CA, USA).

## 3. Results

### 3.1. Characterization of the Compounds through HPLC-PDA Analysis

The analyses were carried out on Shimadzu LC20 AT high performance liquid chromatograph, with a SPDM20A diode array detector and an YMC C30 column (250 × 4.6 mm; 5 μm). The mobile phases consisted in methanol/methyl-tert-butyl ether (MTBE)/water (81:15:4, *v*/*v*/*v*) (solvent A) and MTBE/methanol/water (90:7:3, *v*/*v*/*v*) (solvent B). The gradient was as follows: 0 min, 0% B, 20 min, 0% B; 130 min, 82% B; 132 min, 0% B, followed by equilibration of column for 10 min. The flow rate was fixed at 0.8 mL/min and the DAD, spectra were acquired in the range 300–600 nm the detector being set at 450 nm. Standard compounds zeaxanthin, β-cryptoxanthin and β-carotene were provided by ChromaDex, Los Angeles, CA, USA; zeaxanthin dipalmitate was obtained by semi synthesis and purified by HPLC in our laboratory [39]. The chromatographic data and UV-VIS spectra were compared using Shimadzu LC software. Calibration curves were made with zeaxanthin, β-cryptoxanthin and β-carotene by plotting peak area against concentration for five concentrations ranging from 1–50 μg/mL.

Chromatographic separation of unsaponified carotenoid extract revealed the presence of zeaxanthin esters, non-esterified zeaxanthin and β-carotene. The major compound in the unsaponified extract was zeaxanthin dipalmitate (peak 5 in Supplementary File Figure S1), which represented 83.2% (**1**) and 88.3% (**2**) of total carotenoids (area percentage).

**Figure 1:**
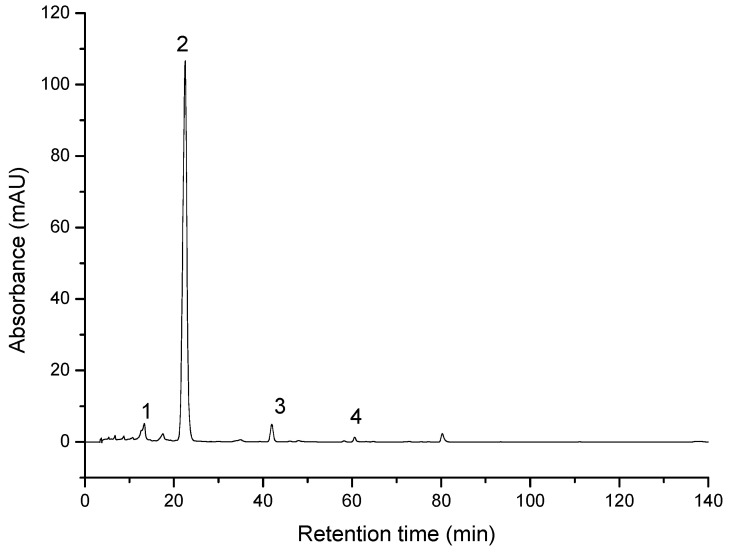
HPLC/PDA chromatogram of saponified Goji berries extract 2: 1-neoxanthin; 2-zeaxanthin; 3-β-cryptoxanthin; 4-β-carotene.

Saponified Goji extracts contained zeaxanthin as the major compound, with 90.2% (sample 1) and 91.16% (sample 2) of total carotenoids). These concentrations were taken into account to normalize the zeaxanthin dipalmitate concentration to 10mM for the biologic testing. In lower amounts were identified β-cryptoxanthin, β-carotene and neoxanthin (Figure 1). The profile and the amount of carotenoids in analyzed samples (Table 1) are in agreement with previously reported data [32,33,40]. The total amount of carotenoids was estimated at 25.99 mg/100 g FW in sample **1** and 26.27 mg/100 g FW in sample **2**.

### 3.2. Evaluation of the Extracts Effect on Skin-Derived Cells Growth

#### 3.2.1. Cytotoxicity

The normal BJ fibroblasts and the A375 malignant melanoma cells were exposed for 24 h to the saponified *Lycium barbarum* (Goji) extracts **1** and **2**, and their cytotoxicity was quantified (Table 2). The one-way ANOVA comparison test indicates no significant differences in IC_50_ values (*p* < 0.05) between **1** and **2** and the extracts cytotoxicity was comparable in BJ and A375 cell lines.

#### 3.2.2. The Antiproliferative Capacity

**Figure 2:**
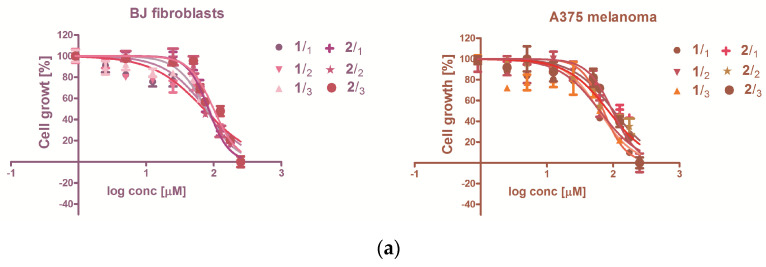

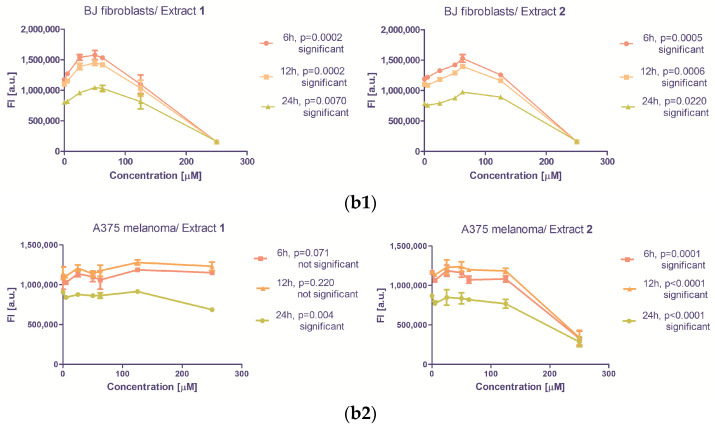
The in vitro effect of zeaxanthin-rich Goji extracts **1** and **2** on the cells survival, and proliferation; (**a**) sigmoidal dose-response relationship between the concentration of 1 and 2 and the inhibition of the cells growth during 24 h of exposure; (**b1**,**b2**) linear dose-response relationship between the compounds concentration and metabolic activity-related Alamar Blue fluorescence (FI on y axis is the fluorescence intensity).

The Alamar Blue fluorescent stain indicates the intracytoplasmic reducing capacity of the cells, implicitly their metabolic status. The comparison between the fluorescence measured in presence or absence of treatment with **1** or **2** reflects their antiproliferative capacity. For this purpose, the modulation of the reducing capacity was assessed with the same concentration range of **1** and **2** was used as for the cytotoxicity testing. The cells’ exposure to zeaxanthin from Goji extracts **1** and **2** caused a decrease in fluorescence, proportionally with the concentration and the exposure time (6, 12 or 24 h, Figure 2, Table 2). In BJ normal cell line and A375 malignant melanoma, extract **2** exhibited antiproliferative activity. The activity of **1** was statistically significant in BJ cells, and in A375 after 24 h of treatment (Figure 2).

The capacity of zeaxanthin from Goji extract to modulate the intracellular reducing potential in treated normal fibroblasts (BJ) and malignant melanoma (A375) population, quantified by linear regression in the 95% confidence interval (Figure 2). The statistical significance of the deviation from untreated cells, considered as concentration 0, was quantified using the hill slope, p being the quantification of the probability. The linear regression in the 95% confidence interval indicates significant decrease following the treatment, exception being the shorter, 6- or 12-h exposure of A375 cells. Considering that the *Lycium barbarum* extracts contain other bioactive compounds as well [41], and the different Goji cultivars display various phenolic patterns [42], they can influence **1** and **2** biologic activity even if the other phenols distribution in **1** and **2** is reduced in comparison with the zeaxanthin.

### 3.3. The Extracts ***1*** and ***2*** Effect on CD44 and CD105 Markers

**Figure 3:**
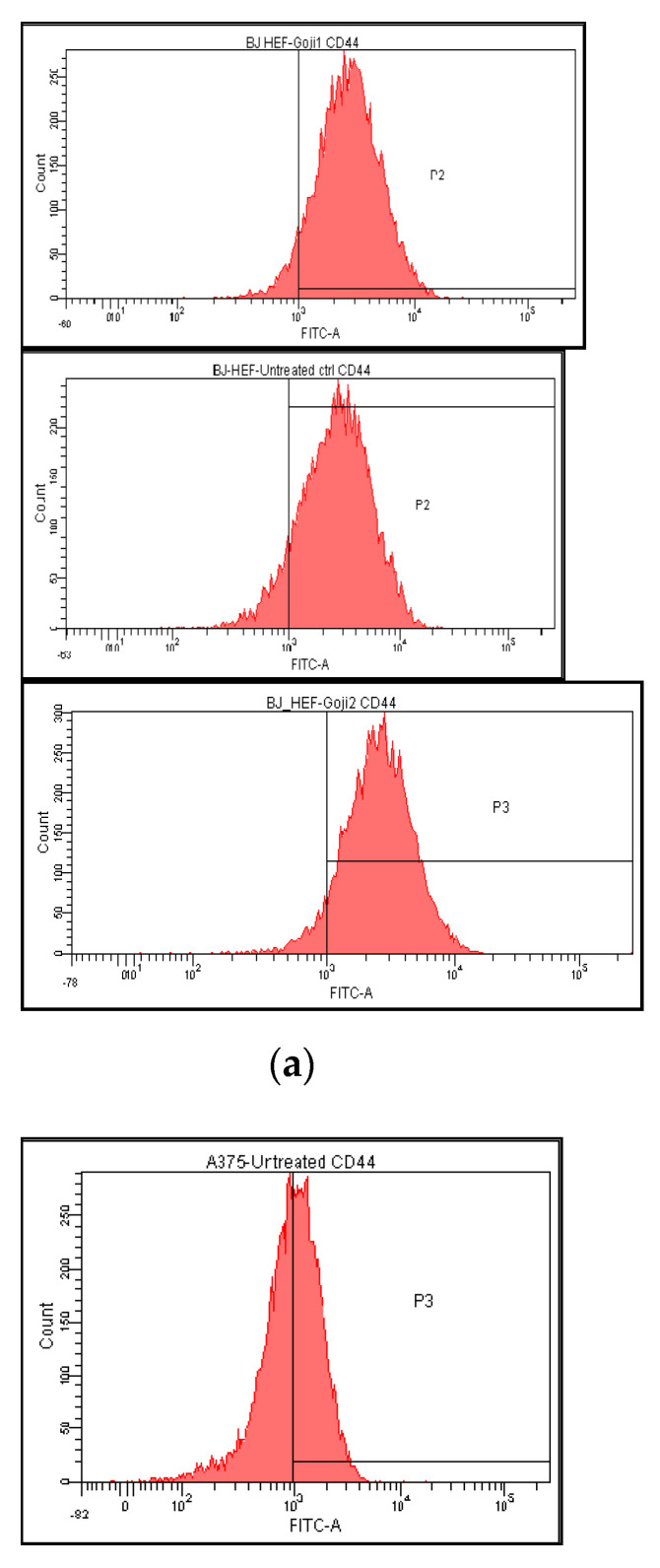

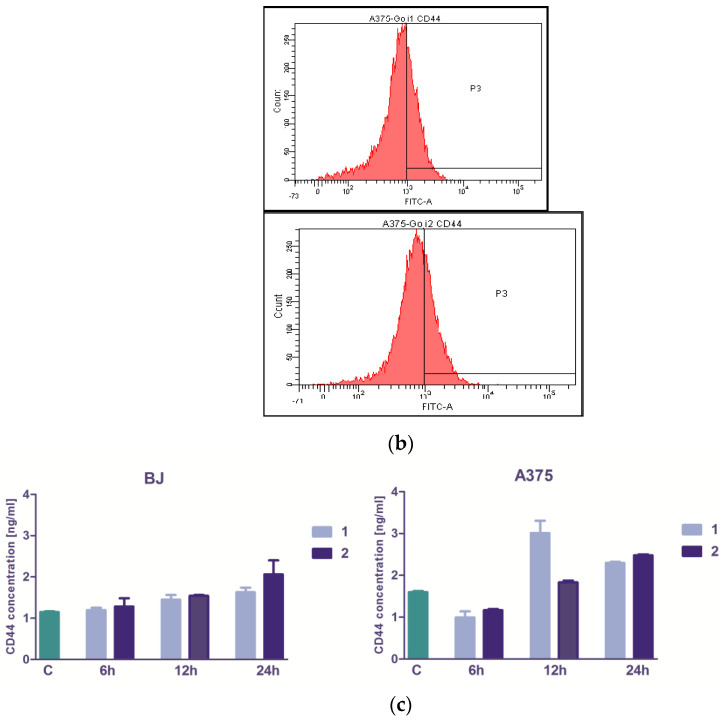
The modulation of CD44 membrane marker expression and the level of secreted CD44 protein in normal BJ versus A375 tumor cells treated with zeaxanthin-rich extract from Goji berries in vitro. The histograms represent the distribution of CD44-positive BJ cells (**a**) and CD44-positive A375 cells (**b**) in untreated control versus treatment with 50μM zeaxanthin-rich extract **1** and **2**, respectively. In the lower row (**c**): the level of secreted CD44 protein by the normal BJ and malignant A375 cells following the same treatments; the columns correspond to the untreated control **C** and zeaxanthin-rich extracts **1** and **2**.

The expression of CD44 membrane marker was abundant on BJ fibroblasts surface (89.51%), and the 24 h exposure to Goji-derived zeaxanthin **1** and **2** generated a slight increase (93.82% and 93.52%, correspondingly, Figure 3 and Table 3). In A375 cells, the untreated reference values were lower, but still 50.13% of the cells were CD44-positive, which decreased significantly (two-way analysis of variance in the 95% confidence interval) following the cells exposure to **1** (to 33.35%) and **2** (to 32.16%). The 24-h exposure to **1** and **2** augmented the secreted CD44 level in A375 cells, dissimilar to the shorter exposures: 6 h and 12 h give inconsistent or statistically insignificant changes. In BJ, only extract **2** was capable to increase the secreted CD44 level, after 24 h exposure (Figure 3), while none of the other exposure lengths was sufficient to significantly modulate CD44 secretion (one-way analysis of variances in the 95% interval).

**Figure 4:**
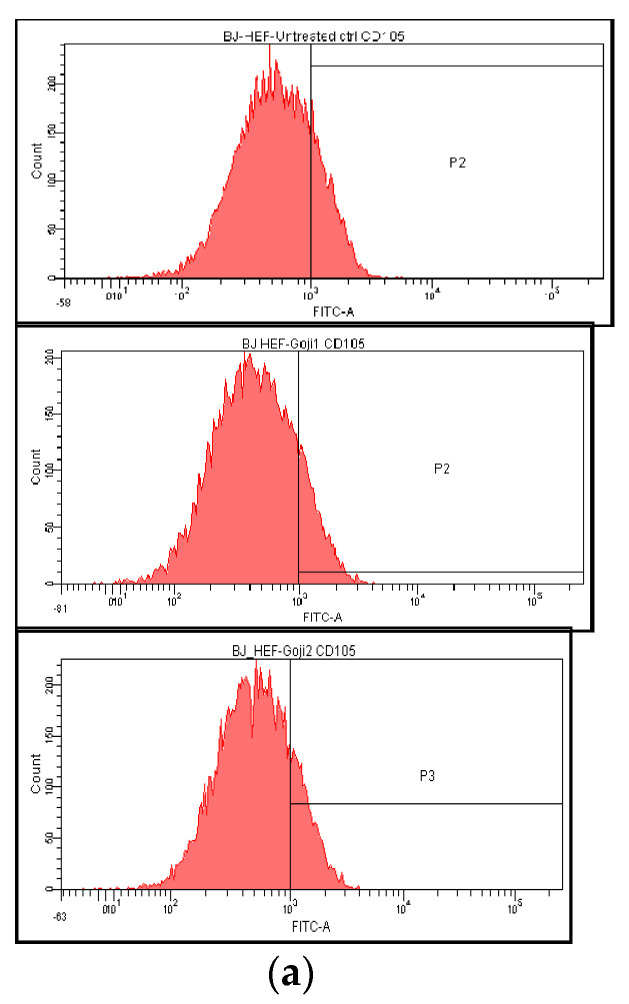

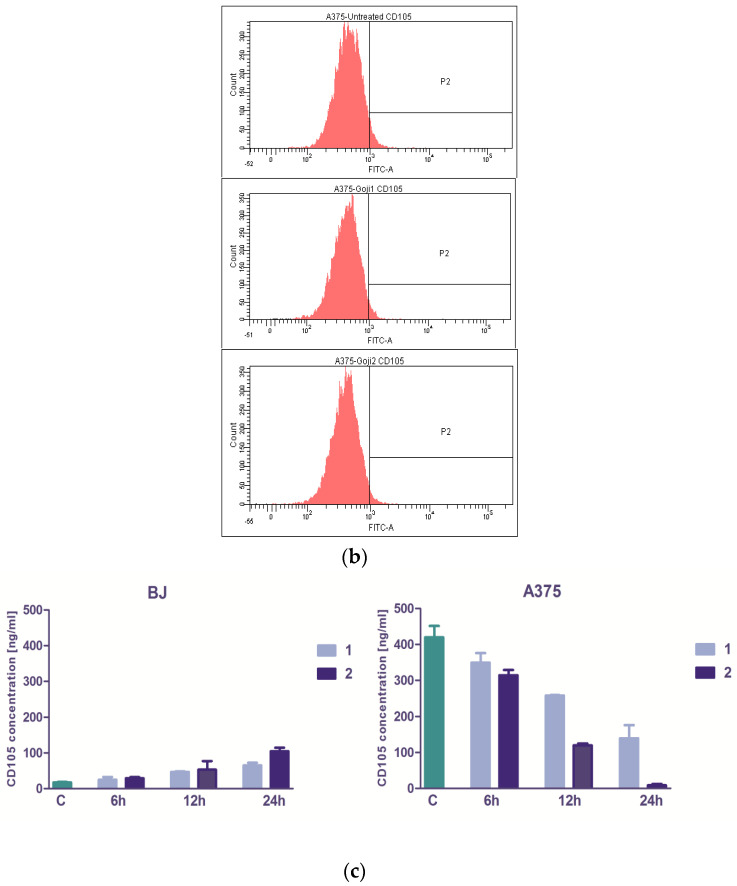
The zeaxanthin-rich extract **1** and **2** influence on CD105 membrane marker expression in normal BJ (**a**) and A375 melanoma cells (**b**). From top down: untreated cells and cells treated for 24 h with 50 μM extracts **1** or **2**. In the lower row (**c**) the secreted soluble CD105 levels are represented in BJ and A375 cells, following the treatment with **1** or **2**, at the same concentration.

The influence of **1** and **2** on CD105-positive cells proportion was less significant (Figure 4). In A375 cells, the CD105 basal value was low (3.99%) and the action of extracts caused insignificant decrease (to 2.70 following **1** and 1.97 following **2** activities). In BJ cells, zeaxanthin **1** solution caused a decrease: from basal 18.83% to 13.72%, while the effect of **2** has had no significance on CD105 marker (17.73%). The cells in vitro CD105 secretor capacity was evaluated with Elisa testing (Figure 4); the A375 melanoma cells CD105 level was significantly higher that of BJ normal fibroblasts, the result being in concordance with previously published data [43]. Extract **2** showed selectivity, acting more efficiently on melanoma cells membranar CD105 (one-way ANOVA, Bonferroni post test, *p* < 0.05) and more, it has had the capacity to stimulate the CD105 production in fibroblast (Figure 4), contrary with the diminishing effect in A375 melanoma.

### 3.4. The Evaluation of NF-kβ Transcription Factor

**Figure 5:**
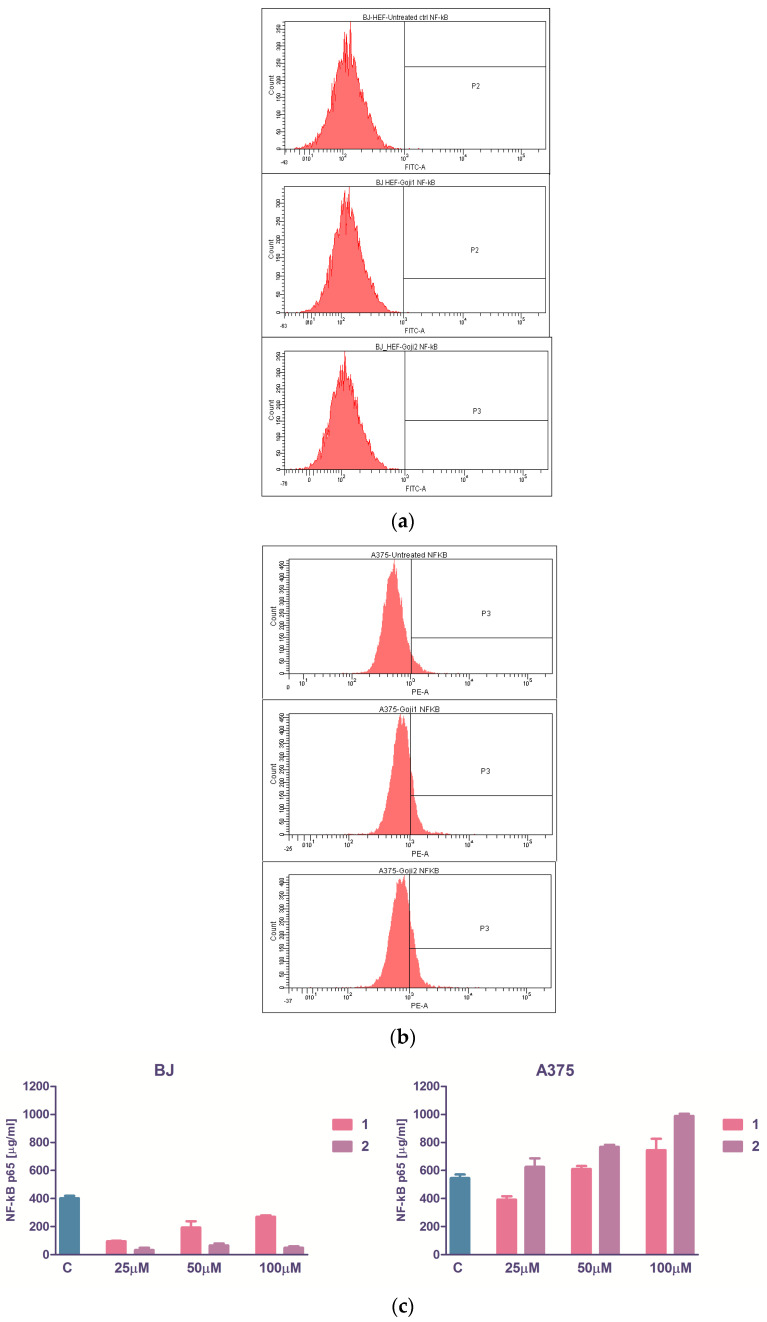
The zeaxanthin-rich extracts influence on intracellular transcription factor NF-kβ p65 is depicted by flow-cytometry generated histograms of normal BJ (**a**) and malignant A375 cells (**b**) subjected to a 24-h treatment; from left to right: untreated cells, treatment with 50 μM **1** and **2**. (**c**) The dose-response relationship between the quantity of zeaxanthin added to the cells and the concentration of the soluble NF-kβ p65 secreted within 24 h.

The soluble form of total p65 NF-kβ was quantified immunoenzymatically with the ELISA testing. The basal values were lower in fibroblasts than in melanoma cells, and the variations in intercellular NF-kβ concentration were not dose-dependent in BJ cells, although a clear tendency of NF-kβ drop was observed, opposite to A375 cells where 50 µM subcytotoxic and 100 µM cytotoxic concentrations both conducted to NF-kβ increase (Figure 5). The flow cytometry showed that the expression of intracytoplasmic phosphorylated form of NF-kβ in BJ cells was nearly zero, and the treatment with 50 µM **1** or **2** does not influenced this molecule. Instead, in A375 cells, the basal activation was higher (Figure 5), which is in concordance with previous findings [44] and the extracts caused a significant increase in NF-kβ: 14.60% versus 5.75% for **1** and 18.50% versus 5.75% for **2**, the tendency of increase being correlated with the soluble NF-kβ for the same treatment (Spearman nonparametric correlation, r 0.94, *p* value 0.0083, very significant).

### 3.5. Modulation of MAPK Signaling

The intracellular MAP kinases signaling were analyzed through flow cytometry, which allowed to quantify the positive cells in treated BJ and A375 populations. Based on the quantitative measurement of the intracellular MAP-kinases JNK, p38 and ERK (Table 3) the percent of up- or down-regulation of the three markers was calculated (Table 4), relative to the untreated cells. In the A375 melanoma, both **1** and **2** zeaxanthin-rich extracts triggered up-regulation of phosphorilated JNK 1/2, ERK1 + ERK2 and p38, while in BJ cells, in most of the cases, the negative values point toward the three intracellular MAP kinases suppression, with one exception: following the exposure to **2**, JNK increased to some extent in compare with the untreated reference.

**Figure 6:**
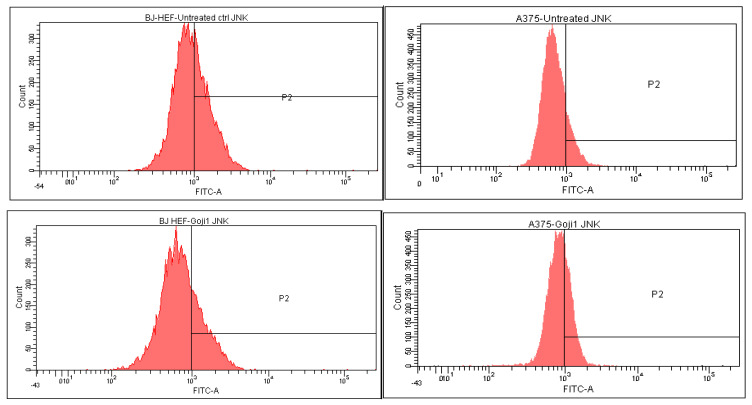

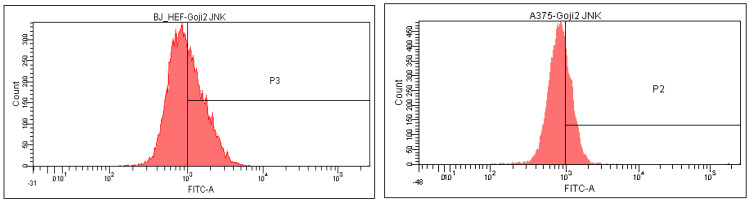
The intracellular c-Jun N-terminal kinases (JNK) modulation in normal BJ fibroblasts versus malignant melanoma A375 cells following the 24-h exposure to zeaxanthin. In the left column, the BJ cells are represented, while in the right column the A375 cells; from top down: cells without treatment (reference), cells treated with **1** and **2**.

In normal BJ fibroblasts, the JNK, ERK and p38 expression was downregulated (Table 4). After the treatment, less positive cells were observed in both BJ and A375; it was a single exception: following the action of zeaxanthin **2**, JNK expression increased with 9.12 percent (40.04% of positive untreated cells versus 43.69% after 24-h incubation with **2**). The largest extent of downregulation was generated by **1** on JNK kinase (Table 4, Figure 6). In malignant A375 cells, the ROS signalling pathways showed a completely different outcome (Table 4), all kinases being strongly upregulated.

**Figure 7:**
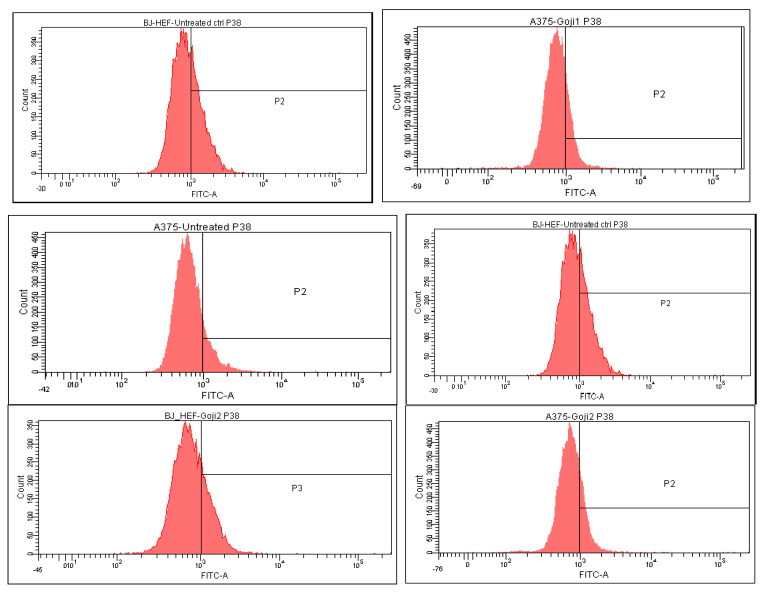
The intracellular p38 expression in normal BJ fibroblasts versus A375 malignant melanoma following the 24-h exposure to zeaxanthin-rich extracts **1** and **2**. In the left column are represented from top down: untreated cells, cells treated with 1, and cells treated with 2, respectively; in the right column, A375 cells, in the same order.

**Figure 8:**
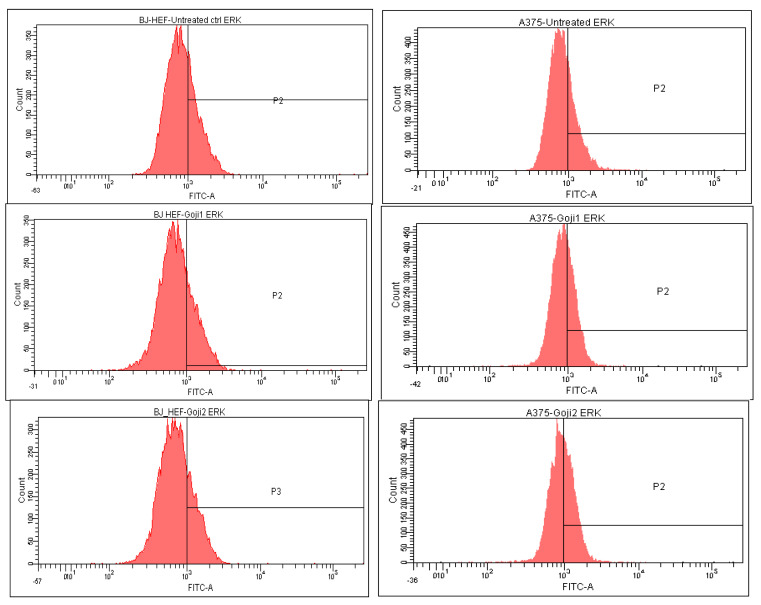
The extracellular signal-regulated kinase ERK 1/2 modulation in normal BJ fibroblasts (**left**) and A375 malignant melanoma (**right**) following the 24-h exposure to zeaxanthin **1** and **2**.

The strongest selectivity was observed as well in the effect of **1** on JNK, the difference between the normal and tumor cells being 146.08% (Figure 6). The distribution of p38-positive cells (Table 4, Figure 7) and the ERK-positive cells (Figure 8) following the 24-h exposure to Goji extracts **1** and **2** showed similarities: in both cases, the percent of positive cells augmented significantly (one-way ANOVA test, *p* < 0.05) in A375 tumor cells, while in normal BJ cells the expression of ERK and p38 was suppressed.

## 4. Discussion and Conclusions

Zeaxanthin dipalmitate was firstly identified in Goji berries by Weller and Breithaupt in 2003 [45], in a study that focused on distribution zeaxanthin esters in various fruit. According to these authors, zeaxanthin represents 89% of the total area of carotenoids (recorded at 450 nm) in the saponified extract, while zeaxanthin dipalmitate was quantified at 160.9 mg/100 g dry material in the unsaponified extract. Later, Inbaraj et al. [36] identified also neoxanthin, β-cryptoxanthin, β-carotene and some of their geometric isomers in the saponified Goji extract. Zeaxanthin represented 1196.8 micrograms/g (89.64% of total carotenoids) in the saponified extract, while zeaxanthin dipalmitate (1143.4 micrograms/g) represented 80.5% in the unsaponified extract. A similar profile was reported [46] for the saponified extract. Environmental factors and processing methods (drying) can affect the total amount of carotenoids in commercial Goji berries.

Zeaxanthin and lutein are xanthophylls (hydroxy carotenoids) that accumulates preferentially in the human retina where they seem to have a protective effect by acting as filter pigments or as antioxidants [47]. Zeaxanthin is present in fruit and vegetables in both free and esterified form. While corn or egg yolk are good sources of free zeaxanthin, Goji berries, sea buckthorn berries or orange pepper are rich in zeaxanthin mono- and diesters [48]. Supplementation of human subjects with Goji berries resulted in a significant increase of free zeaxanthin (but not that of esterified zeaxanthin) in plasma, demonstrating that esters are efficiently hydrolyzed and absorbed [45,49]. Very low concentrations of xanthophylls esters were found in plasma or human skin after long-time supplementation with high doses of esters [50,51]. Chitchumroonchokchai and Failla [52] showed that zeaxanthin esters were partially hydrolyzed by carboxyl ester lipase and free zeaxanthin was the most abundant form in Caco-2 cells. All these experimental data sustain the hypothesis of the complete hydrolysis of ester fraction during digestion and a postabsorptive acylation of xanthophylls. Considering these observations, in the present study we decided to use the saponified extract of Goji berry.

The zeaxanthin-rich extracts from Goji Erma (1) and Biglifeberry (2) inhibited at some extent the fibroblast proliferation in vitro, these results being in accordance with previous studies [7]. Zeaxanthin effect was dissimilar to those of glycoconjugates from Goji, which promoted the survival of human fibroblasts [20].

The activation of NF-kβ can be quantified evaluating the extent of its nuclear p65 subunit translocation [39]. NF-kβ act like a molecular switch in melanoma [44]. In late stages of melanoma, it is activated and inhibits the apoptosis, and is therefore associated with tumor progression; thus, in early stages, NF-κB upregulates the caspase-dependent apoptotic pathways. The upregulation of the NF-kβ indicates cancer progression, and many natural products were identified as blockers of this signaling pathway [39]. In the case of *L. barbarum* extracts **1** and **2**, no inhibition of p65 intracytoplasmic translocation was observed in A375 cells (Figure 5), while in uveal melanoma the inhibition of NF-kB pathway was reported [7].

CD105 is a transforming growth factor TGF-β co-receptor, its interaction with TGF-β1 inhibits the TGF-β mediated ERK signaling [53], resulting in decreased endothelial cell migration and cell adhesion, and influences the apoptosis and proliferation in many cell types, including skin cells. CD105 targeting through natural compounds such zeaxanthin could be a promising therapeutic approach for malignant melanoma [54] and the disruption of membrane endoglin can suppress the tumor progression [55]. Since zeaxanthin-rich Goji extracts **1** and **2** has had the ability to reduce both membrane CD105 and especially the soluble CD105 in malignant melanoma cells, it could be of interest in new drugs development.

Unlike previous studies, the intracytoplasmatic expression of MAP kinases was assessed with flow cytometry methods at unicellular level. In melanoma cell the ERK-, JNK- and p38-mediated signaling pathway is involved in melanin production as well [30]. Zeaxanthin-rich extracts **1** and **2** act as p38 suppressors in normal skin cells, same as other fractions extracted from L. barbarum [4]; in A375 melanoma cells, the opposite effect was observed. The three MAP kinases were significantly upregulated by **1** and **2** in A375 cells. The activation of JNK displays versatile effects on malignant melanoma cells. Wang and co-authors [56] reported a correlation between JNK activation and the tumor proliferation in vivo in mice, while other studies indicated that JNK activation by nutraceuticals trigger apoptosis in melanoma cells [57]. The mitogen activated protein kinases (MAPK) are therapeutic targets in cancer [58]. ERK is involved in cell survival and proliferation and can activate transcription factors such NF-κB; therefore, the overexpression of ERK 1/2 point towards the proapoptotic processes in A375 cells. The increase of phosphorilated JNK, ERK1/2 or p38 membrane expression could lead to an augmented pro-oxidative process inside the tumor cells, detrimental to the survival of tumor cells. Zeaxanthin was able to regulate the increase of JNK and p38 expression in gastric tumor cells through the increase of ROS levels [59], and this confirms our hypothesis. Overall, the effect of **1** and **2** on A375 melanoma cells in vitro raises questions about the opportunity of their employment as antitumor agents, and downstream effects of these pathways need to be explored as well.

The fact that **1** and **2** have had similar, but not identical, effect denotes that besides zeaxanthin, the rest of the components (under 17%, and 12% in the composition of extracts) may play an important biologic role as well, and the plant cultivar marked its own biologic fingerprint on human skin cells in vitro, which can be extrapolated to in vivo effect.

Extracts **1** and **2** showed a beneficial cytoprotective effect on normal skin fibroblasts at subcytotoxic concentrations, the threshold of 50 µM being optimal for in vitro applications. For a possible dietary intake of zeaxanthin or topical applications on skin, the recommended concentration has to be tested in vivo.

In vitro, the zeaxanthin-rich Goji extracts **1** and **2** have had a moderate activity against A375 melanoma cells, and there are no major differences between their toxicity on normal and tumor cells. Moreover, the JNK, ERK, p38 and the total NF-kβ were upregulated, and these results point out towards an antiapoptotic pattern, even if they are able to reduce the tumor cells growth. However, the distribution of CD44-positive and CD105-positive cells after 1 and 2 treatment clearly indicated the reduction of stemness, induction of proapoptotic signaling in melanoma cells. Therefore, we can suppose that, in vivo, **1** and **2** might be efficient alone as prodrugs but more likely as adjuvants to the standard antitumor chemotherapy, but this needs further investigation.

## Figures and Tables

**Table 1:** Carotenoids composition (mg/100 g FW +/− SD) in the saponified Goji extracts.

ID	Compound	Retention Time	UV-Vis Maxima	Sample 1 mg/100 g FW	Sample 2 mg/100 g FW
1	Neoxanthin	14.6	416, 439, 468	0.70 ± 0.14	0.75 ± 0.16
2	Zeaxanthin	22.1	425, 450, 476	23.44 ± 1.02	23.69 ± 1.13
3	β-cryptoxanthin	41.9	425, 451, 476	0.60 ± 0.15	0.49 ± 0.14
4	β-carotene	60.6	421, 452, 477	0.07 ± 0.02	0.07 ± 0.02

**Table 2:** The cytotoxicity of zeaxanthin-rich extracts **1** and **2,** expressed as half inhibitory concentration (IC_50_, sigmoidal dose-response) and the antiproliferative capacity expressed as significant negative hillslope derived from the linear regression of time-dependent inhibition.

		IC_50_ Values (μM)			
Cell Line		BJ (CRL-2522)		A375	
**1**	24 h	75.15 ± 0.23		62.36 ± 0.08	
**2**	24 h	85.06 ± 11.34		92.59 ± 6.71	
		**Inhibition of Proliferation**			
**Cell line**		**BJ (CRL-2522)**		**A375**	
		Hillslope	*p* value	Hillslope	*p* value
**1**	6 h	−4726 ± 905.0	0.0002	459.6 ± 228.7	0.0675
	12 h	−4274 ± 822.5	0.0002	389.2 ± 301.3	0.2208
	24 h	−2754 ± 606.8	0.0007	−654.8 ± 182.8	0.0038
**2**	6 h	−4179 ± 877.0	0.0005	−3166 ± 576.8	0.0001
	12 h	−3706 ± 807.5	0.0006	−3076 ± 482.9	<0.0001
	24 h	−2253 ± 582.5	0.0022	−2109 ± 345.0	<0.0001

**Table 3:** Regulation of membrane- and intracellular marker expression in tumor and normal cells in vitro by the treatment with Goji-derived zeaxanthin **1** and **2**.

Cells and Treatments	CD44	CD105	NF-kβ	JNK	p38	ERK
**A375**						
Untreated	50.1 ± 0.40	4.0 ± 0.02	5.8 ± 0.05	14.3 ± 0.03	14.1 ± 0.01	29.2 ± 0.11
Extract 1	33.4 ± 0.16	2.7 ± 0.01	14.6 ± 0.02	30.7 ± 0.05	19.4 ± 0.08	34.5 ± 0.14
Extract 2	32.2 ± 0.18	2.0 ± 0.00	18.5 ± 0.12	29.3 ± 0.15	20.0 ± 0.04	42.4 ± 0.27
**BJ**						
Untreated	89.5 ± 0.10	18.8 ± 0.03	0.02 ± 0.00	40.0 ± 0.14	35.4 ± 0.25	30.9 ± 0.13
Extract 1	93.8 ± 0.02	13.7 ± 0.12	0.01 ± 0.00	27.4 ± 0.05	32.3 ± 0.04	25.9 ± 0.07
Extract 2	93.5 ± 0.05	17.7 ± 0.07	0.05 ± 0.00	43.7 ± 0.11	25.4 ± 0.02	25.1 ± 0.06

**Table 4:** The influence of zeaxanthin-rich Goji extracts **1** and **2** on phosphorilated mitogen activated protein (MAP) kinases-driven ROS signaling upregulation (depicted as ↑) or downregulation (depicted as ↓) in normal and malignant cells in vitro.

Mitogen Activated Protein Kinases (MAPK)	Cell Line	BJ		A375	
	Treatment	1	2	1	2
JNK		↓ 31.68%	↑ 9.12%	↑ 114.40%	↑ 104.61%
ERK		↓ 16.02%	↓ 18.72%	↑ 18.28%	↑ 45.42%
p38		↓ 9.00%	↓ 28.41%	↑ 36.75%	↑ 41.63%

## Data Availability

Not applicable.

## Supplementary Materials

Figure S1: The major compound in the unsaponified extract was zeaxanthin dipalmitate (peak 5).

## Sample Availability

Samples of the compounds are not available from the authors.
