# Supplementary figures and images for: Zeaxanthin-Rich Extract from Superfood Lycium barbarum Selectively Modulates the Cellular Adhesion and MAPK Signaling in Melanoma versus Normal Skin Cells In Vitro
Source: Molecules. 2021 Jan 11;26(2):333. doi: 10.3390/molecules26020333 (PMC7827977; doi:10.3390/molecules26020333)

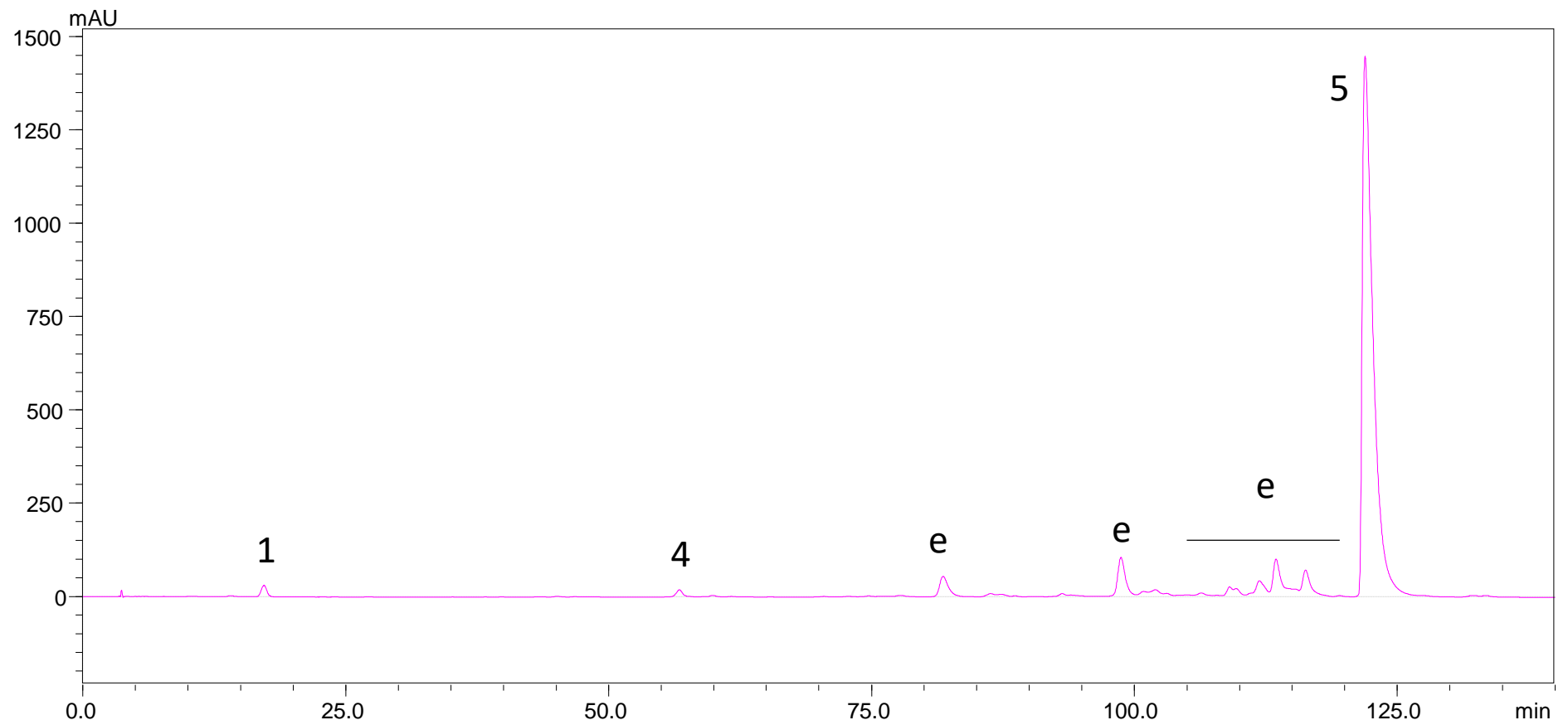

Figure S1. The major compound in the unsaponified extract was zeaxanthin dipalmitate (peak 5 )

Supplement: Supplementary file 1 [file molecules-26-00333-s001.pdf]
